# A Cup of Tea

**Published:** 1881-01

**Authors:** 


### A CUP OF TEA.

In a recent lecture by Mr. R. G. Tweedie, F. C. S., London, on "A Cup of Tea," the speaker divided his subject into four sections—the tea, the water, the milk and the sugar. The lecturer first drew attention to tea drinking with every-day life, and showed that the principal components of tea were theine and the essential oil of tannin, which latter possessed astringent properties. He informed the audience that the best time to take tea was about three hours after dinner or any other heavy meal, and deprecated in the strongest terms the excess to which tea drinking is carried by some people, asserting that such a practice induced a nervous disorganization and impeded digestion. He showed that the sole difference between black and green tea was one of preparation and that both kinds could be obtained from the leaves of the same plant. After asserting that the adulteration of tea had very much decreased of late years, which the tea drinking public will be glad to know, the lecturer proceeded to treat of the various kinds of shrubs grown in different parts of the world and the countries where the different kinds of teas were consumed, the lecturer came to the consideration of the milk, its value as a nutritive agent, and referring to its adulteration he made the astounding assertion that in London alone every year no less than £70,000 was spent on water which was sold as milk. Passing on to regard the sugar, the lecturer denied the common error that sugar was injurious to the teeth, bringing forward as an example the negroes of Jamaica, who, he said, though they were the greatest eaters of sugar in the world, were proverbial for their beautiful teeth.

